# Sharing Patient and Clinician Experiences of Moderate-to-Severe Psoriasis: A Nationwide Italian Survey and Expert Opinion to Explore Barriers Impacting upon Patient Wellbeing

**DOI:** 10.3390/jcm11102801

**Published:** 2022-05-16

**Authors:** Francesca Prignano, Alexandra M. G. Brunasso, Gabriella Fabbrocini, Giuseppe Argenziano, Federico Bardazzi, Riccardo G. Borroni, Martina Burlando, Anna Elisabetta Cagni, Elena Campione, Elisa Cinotti, Aldo Cuccia, Stefano Dastoli, Rocco De Pasquale, Clara De Simone, Vito Di Lernia, Valentina Dini, Maria Concetta Fargnoli, Elisa Faure, Alfredo Giacchetti, Claudia Giofrè, Giampiero Girolomoni, Claudia Lasagni, Serena Lembo, Francesco Loconsole, Maria Antonia Montesu, Paolo Pella, Paolo Pigatto, Antonio Giovanni Richetta, Elena Stroppiana, Marina Venturini, Leonardo Zichichi, Stefano Piaserico

**Affiliations:** 1 Department of Health Sciences, Section of Dermatology, University of Florence, 50125 Florence, Italy; 2 Department of Dermatology, Villa Scassi Hospital-ASL 3, 16149 Genova, Italy; giovanna.brunasso@gmail.com; 3 Section of Dermatology, Department of Clinical, Medicine and Surgery, University of Naples Federico Ii, 80138 Naples, Italy; gafabbro@unina.it; 4 Dermatology Unit, University of Campania, 80138 Naples, Italy; g.argenziano@gmail.com; 5 Dermatology Unit—IRCCS Policlinico di Sant’Orsola, Department of Experimental, Diagnostic and Specialty Medicine, University of Bologna, 40126 Bologna, Italy; federico.bardazzi@aosp.bo.it; 6 Dermatology Unit, Humanitas Clinical and Research Center—IRCCS, Department of Biomedical Sciences, Humanitas University, 20090 Milan, Italy; riccardoborroni@gmail.com; 7 Dermatologic Clinic, DISSAL, San Martino Policlinico San Martino Hospital, 16132 Genova, Italy; martinaburlando@hotmail.com; 8 Unità Operativa Dipartimentale di Dermatologia e Venereologia, Ospedale San Gerardo—Monza, 20900 Milan, Italy; a.cagni@asst-monza.it; 9 Dermatologic Unit, Department of Systems Medicine, University of Rome Tor Vergata, 00133 Rome, Italy; campioneelena@hotmail.com; 10 Dermatology Unit, Department of Medical, Surgical and Neurological Sciences, University of Siena, 53100 Siena, Italy; elisacinotti@gmail.com; 11 Unit of Dermatology, San Donato Hospital, 52100 Arezzo, Italy; dott.aldocuccia@gmail.com; 12 Department of Health Sciences, Magna Graecia University of Catanzaro, 88100 Catanzaro, Italy; stefanodastoli@tiscali.it; 13 Dermatology Unit, San Marco Hospital, 95123 Catania, Italy; r.depasquale@unict.it; 14 Institute of Dermatology, Catholic University, 00185 Rome, Italy; clara.desimone@unicatt.it; 15 Dermatology Unit, Fondazione Policlinico Universitario A. Gemelli IRCCS, 00168 Rome, Italy; 16 Dermatology Unit, Arcispedale S. Maria Nuova, Azienda USL-IRCCS di Reggio Emilia, 42122 Reggio Emilia, Italy; dilernia.vito@ausl.re.it; 17 Unit of Dermatology, University of Pisa, 56126 Pisa, Italy; valentinadini74@gmail.com; 18 Dermatology, Department of Biotechnological and Applied Clinical Sciences, University of L’Aquila, 67100 L’Aquila, Italy; mariaconcetta.fargnoli@univaq.it; 19 Dermatology Unit, Fondazione IRCCS Ca’ Granda, Ospedale Maggiore Policlinico, University of Milan, 20122 Milan, Italy; elisa.faure87@gmail.com; 20 UOC Dermatology, IRCCS INRCA, 60124 Ancona, Italy; alfredogiacchetti@gmail.com; 21 Dermatology Complex Operative Unit, Papardo Hospital, 98158 Messina, Italy; claudiagiofre@tiscali.it; 22 Section of Dermatology and Venereology, Department of Medicine, University of Verona, 37129 Verona, Italy; giampiero.girolomoni@univr.it; 23 Dermatology Unit, Surgical, Medical, and Dental Department of Morphological Sciences Related to Transplant, Oncology and Regenerative Medicine, University of Modena and Reggio Emilia, 41121 Modena, Italy; lasacla65@gmail.com; 24 Department of Medicine, Surgery and Dentistry, “Scuola Medica Salernitana”, University of Salerno, 84084 Fisciano, Italy; slembo@unisa.it; 25 Department of Biomedical Sciences and Human Oncology, University of Bari Aldo Moro, 70121 Bari, Italy; franciscus59@gmail.com; 26 Azienda Ospedaliero Universitaria Consorziale Policlinico di Bari, 70124 Bari, Italy; 27 Department of Surgical, Microsurgical and Medical Sciences, Dermatology, University of Sassari, 07100 Sassari, Italy; mmontesu@uniss.it; 28 Dermatologia, Ospedale degli Infermi, 13875 Biella, Italy; paolo.pella@aslbi.piemonte.it; 29 Clinical Dermatology, Department of Biomedical, Surgical and Dental Sciences, Istituto Ortopedico Galezzi, University of Milan, 20122 Milan, Italy; paolo.pigatto@unimi.it; 30 Unit of Dermatology, Department of Internal Medicine and Medical Specialties, Sapienza University of Rome, 00185 Rome, Italy; antonio.richetta@uniroma1.it; 31 Section of Dermatology, Department of Medical Sciences, University of Turin, 10124 Turin, Italy; elena.stroppiana@gmail.com; 32 Dermatology Department, University of Brescia, ASST Spedali Civili, 25121 Brescia, Italy; marina.venturini@unibs.it; 33 Unit of Dermatology, San Antonio Abate Hospital, 80057 Trapani, Italy; dermatologia@asptrapani.it; 34 Dermatology Unit, Department of Medicine, University of Padova, 35122 Padova, Italy; stefano.piaserico@gmail.com

**Keywords:** psoriasis, perspective, health-related quality of life, wellbeing, patients, physicians, surveys and questionnaires

## Abstract

A nationwide survey was conducted in adult patients with psoriasis (PsO) across Italy to obtain their real-world perspective of the impact of PsO on their wellbeing. Patients completed a 26-question survey (based on the patient benefit index; PBI, The Dermatology Life Quality Index; DLQI and the World Health Organization-five; WHO-5 wellbeing index) and workshop discussion sessions were undertaken by dermatologists to interpret results from the survey. 392 patients with PsO completed the survey. Analysis of results was restricted to patients who had moderate-to-severe plaque psoriasis (assessed by patients; *n* = 252; 64.3%). Dermatologists (*n* = 32) completed one question from the survey related to wellbeing and rated social, physical and mental domains as contributing to a similar extent, with comparable scores also observed by patients. For treatment, biologics yielded higher scores on average, whereas little difference was observed between topical and conventional systemic treatments. Only 23.8% of patients felt that their dermatologist was taking into consideration their wellbeing and 32.6% of the patients considered their therapy as inadequate in improving signs and symptoms of the disease. This survey identified key factors contributing to barriers impacting on patient wellbeing. Simple, but comprehensive questionnaires can provide important insight to patients’ needs that may significantly increase clinician awareness during visits leading to tailored treatment.

## 1. Introduction

Psoriasis is a chronic, inflammatory immune skin disease affecting ~2% of individuals worldwide [1] associated with physical disability, reduced psychological wellbeing and impaired quality of life (QoL) [2,3,4].

Severity of psoriasis is generally assessed using the psoriasis area severity index (PASI), body surface area (BSA) or Physician Global Assessment (PGA) [5,6], while Patient’s QoL is assessed using the Dermatology Life Quality Index (DLQI) or the Short Form (SF-36) Health Survey [7].

The availability of biological agents, in particular, novel interleukins (IL) such as IL-17 and IL-23 inhibitors, has allowed dermatologists to successfully treat moderate-to-severe psoriasis, with many patients achieving clear skin [8,9,10,11,12,13] and improving their QoL [14,15,16]. However, many patients (e.g., moderate-to-severe psoriasis) may still be untreated/undertreated, decline or fail to respond to or experience side effects [17,18].

Psoriasis can significantly impact upon patients self-image, leading to embarrassment due to visible lesions, consequently resulting in low self-esteem, anxiety and depressive symptoms [19,20,21,22]. In this regard, the impact of psoriasis goes beyond the obvious severity of skin lesions, as demonstrated by the discordance between the QoL scores (e.g., DLQI) and clinical severity (i.e., PASI) [23,24].

The social and psychological impact of psoriasis is generally underestimated by healthcare professionals [25,26,27] and endpoints used in clinical studies do not capture the full impact of this condition [28]. Therefore, the perception of QoL is considered a critical measure in dermatology [29,30] and there is an urgent need to refine QoL measures to identify issues not frequently included in QoL instruments in clinical practice.

To address this unmet need, a nationwide survey was undertaken involving dermatologists across Italy to gather information from the perspective of dermatologist and patient to identify key barriers to be overcome to improve the QoL in these patients.

## 2. Materials and Methods

### 2.1. Study Design

SHAPE (SHAring Patient Experiences) was a prospective cross-sectional nationwide survey conducted in adult patients with chronic plaque psoriasis involving 32 dermatologists (from 32 centers) divided into 4 groups by macroarea (North West, North East, Centre and South Italy).

**Figure 1:**
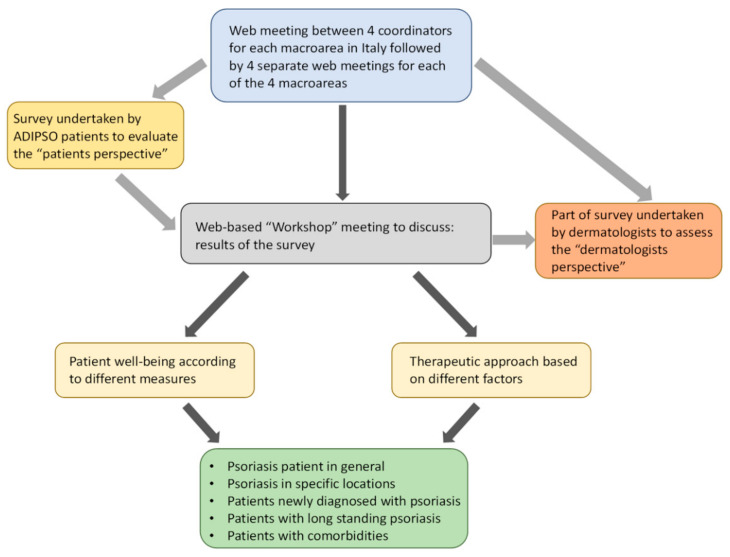
Flow chart of different stages undertaken before and during implementation of the online patient survey.

All patients were members of ADIPSO (*Associazione per la Difesa degli Psoriasici*; Italian association for the defence against psoriasis) [25]. Web-based meetings were held between the 4 coordinators (FP, AMGB, GF and SP) of the 4 macroareas to discuss the design and implementation of the survey. Patients then completed the online survey (Figure 1).

**Figure 2:**
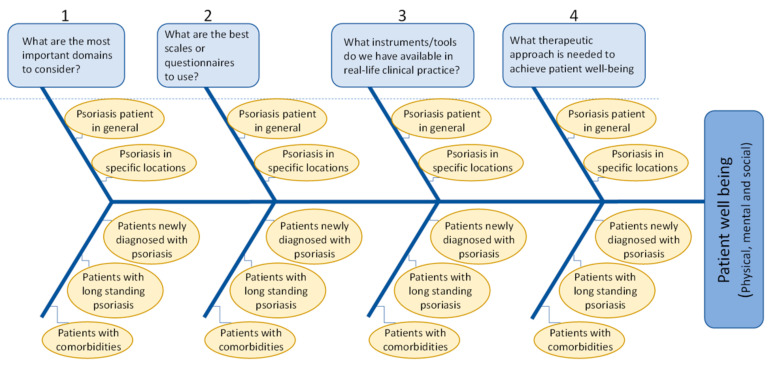
Ishikawa diagram mimics a fish skeleton and summarising the main factors influencing patient wellbeing [26]. The underlying problem (achievement of patient wellbeing) is placed as the fish’s head and the causes/factors extend to the left (numbered 1–4) as the bones of the skeleton; the ribs branch off the back and denote major causes (5 variables for each of the 4 main domains).

All patients had at least primary level education, the majority (86.2%) having secondary or third-level education and therefore deemed capable of reading and understanding the questionnaire prior to being enrolled. Separate web-based meetings were also undertaken for each macroarea to monitor status of the completion of the survey and address any unresolved issues. During these meetings, all dermatologists completed a part of the patient survey to rate how social, physical and mental components contribute to patient wellbeing (Question 23 of the survey; Supplementary Materials S1). A further two web-based meetings were undertaken, involving the 4 coordinators to discuss the results from the patient survey and subsequently with all dermatologists. During these web-based meetings, workshop discussion sessions were also undertaken by dermatologists (with the aid of an Ishikawa fishbone diagram) [26] to interpret results from patients, in order to identify and overcome/provide solutions to unmet needs (Figure 2).

### 2.2. Dermatologists

All 32 dermatologists have extensive experience in the management of patients with psoriasis and a particular interest in the wellbeing and QoL of patients with this disease according to their publication record, participation in conferences, clinical trials and consensus statements and/or senior academic rank.

### 2.3. Online Survey

An online survey was developed by the 4 coordinators and was based on the patient benefit index; PBI [27], the DLQI [7,31] and the World health Organization; WHO-5 wellbeing index [32,33]. Patients were prospectively asked to complete the survey across the 4 macro areas that was accessed through the dedicated ADIPSO website: http://www.adipso.org/sito/it/ (accessed on 12 November 2021) and included 26 specific questions relating to socio-demographic information (Q:1–7) and information relating to their psoriasis disease (Q:8–22) in addition to questions related to patient’s wellbeing (Q23–26) that could be completed in <10 min (Supplementary Materials S1). Question 23 was based on the PBI questionnaire whereas. The first 5 statements of Q24 were based on the WHO-5 and statements 6–12 were based on DLQI and PBI. Q25 and Q26 were developed by the Authors. Additional information on specific aspects relating to the development of this questionnaire are summarised in Supplementary Materials S2. All data collected through this survey was derived from the patient (i.e., disease-severity was assessed by the patient). Patients who could not complete the online survey (for whatever reason) could contact ADIPSO for assistance to complete the survey. Upon completion of the questionnaire, patients gave their consent to the processing of data. Data were collected in an anonymous and aggregated form in compliance with the provisions of art. 13 of the RGPD (EU) 2016/679. The original survey is in Italian language and a translated version in English language is available.

### 2.4. Statistical Analysis

Descriptive statistics were used to summarise clinical characteristics (mean ± SD or number and %). Scores for some variables are presented as box-whisker plots showing median and interquartile range. Comparisons between characteristics of patients with mild disease vs. moderate-to-severe psoriasis were performed by the Fisher’s exact test for categorical variables and the Wilcoxon test for non-parametric continuous variables. Comparison between scores for the three types of treatment (biological, systemic or topical) was performed by 1-way ANOVA followed by Bonferroni post-hoc. Data derived from the online survey are summarised as number and %. A *p*-value of ≤ 0.05 was considered statistically significant and analysis was performed using MedCalc software (Mariakerke, Belgium).

## 3. Results

### 3.1. Patient Clinical Characteristics

A total of 392 patients with PsO participated in this multiregional survey. Patient clinical and demographic characteristics are summarised in Table 1. The majority of patients were male (53.6%) aged 52.4 ± 14.8 years and had a long history of PsO (disease duration of 22.5 ± 14 years). Approximately 85% of patients completed secondary school or had a university degree and 56.1% of them were currently employed. Stratifying patients by PsO severity, some significant differences emerged. Patients with moderate-to-severe PsO (assessed by the patients) were significantly older (53.7 ± 13.5 vs. 49.01 ± 17.5 years, *p* = 0.017), had a higher BMI (26.5 ± 4.4 vs. 25.1 ± 4.3 kg/m^2^, *p* = 0.015) and a lower proportion were students (2.8 vs. 10.9%, *p* = 0.009). Lesions were mainly localised to the elbow/knee (70.1%), scalp (63.7%) or chest (50%) and the most frequent comorbid diseases were hypertension (27.7%) and rheumatological disease (25.7%). The frequency of lesions localised at the chest and hands/feet and nails was higher in moderate-to-severe patients and these patients also presented a higher (approximately two-fold) burden of comorbid diseases such as joint disease (27.4 vs. 15.1%, *p* = 0.046).

### 3.2. Previous and Current Treatment

The majority of patients were previously treated with topical medication (75.9%), a higher proportion of patients with moderate-to-severe disease having received ≥1 conventional systemic therapy compared to patients with mild disease (40.9 vs. 9.6%, *p* < 0.0001) (Table 2). Regarding current treatment, about one-third of patients were currently receiving topical treatment (34.6%), with a significantly higher proportion of patients with mild disease (64.4 vs. 27.8%, *p* < 0.0001). In contrast, a higher proportion of patients with moderate-to-severe disease were receiving biological treatment compared to those with mild disease (42.5 vs. 4.1%, *p* < 0.0001). Thirty-four patients (13.5%) with moderate-to-severe PsO were not currently receiving any treatment.

### 3.3. Impact of PsO Treatment on Signs and Symptoms, QoL and Impact in Workplace

For the present analysis, data analysed from the survey were restricted to patients with moderate-to-severe plaque PsO, accounting for 252 (64.3%) of all patients.

Considering all patients with moderate-to-severe PsO (*n* = 252), 32.6% of patients felt that their treatment was poor or bad with regard to improving signs/symptoms of the disease (Table 3). Stratifying by treatment type revealed that as many as 80% of patients receiving biological treatment thought their treatment was “great” or “good” compared to only 12.5% of patients receiving conventional systemic treatment or 18.6% of patients receiving topical treatment. This trend was also seen when patients were asked to rate their treatment in terms of QoL and impact in the workplace; poorest for systemic therapy, followed by topical and greatest satisfaction seen with biological treatment (Table 3).

### 3.4. Perspective of How Patients Think Aspects Related to Use of Questionnaires and How Wellbeing Is Considered by Their Dermatologist

**Figure 3:**
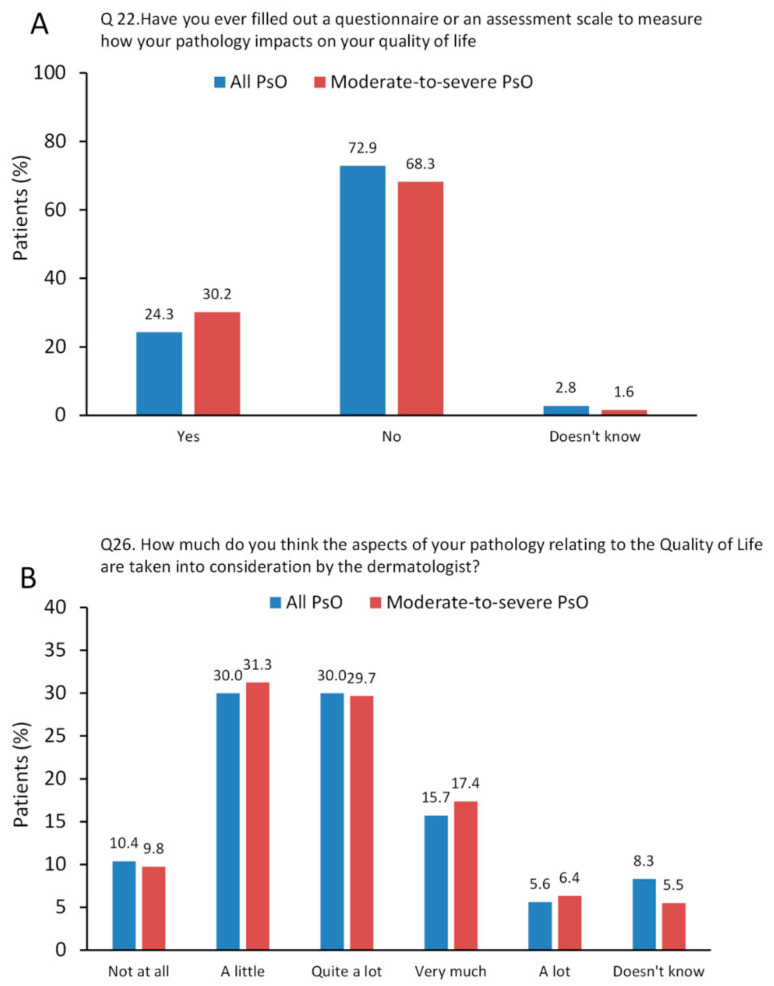
Results from questions/statements related to Questions no. 22 and 26 from the online questionnaire. (**A**), Q22: Have you ever filled out a questionnaire or an assessment scale to measure how your pathology impacts on your quality of life (e.g., daily activities that you are more or less able to carry out, impact of the disease on social relationships, psychological distress, …)? (choose from yes or no, doesn’t know/doesn’t answer); (**B**), Q26: How much do you think the aspects of your pathology relating to the Quality of Life (work environment, social relationships, psychological state, etc.) are taken into consideration by the dermatologist? (choose from; not at all, a little, quite a lot, very much, a lot, doesn’t know how to respond or doesn’t respond). Data are presented as %.

Only 24.3% of patients recall taking a QOL questionnaire to measure how their disease impacts on their QoL (Q:22 of the online survey; Supplementary Materials S1) and the proportion of patients having previously completed a questionnaire increased slightly to 30.2% in patients with moderate-to-severe PsO (Figure 3A). Approximately 24% of patients thought that their disease related to QoL had been taken into consideration (“very much” or “a lot”) by their dermatologist (Q:26 from the online survey) and about 40% of patients responded “not at all” or “a little” (Figure 3B).

### 3.5. Perspective from Dermatologists on Physical, Social and Psychological Domains Compared to Patients

**Figure 4:**
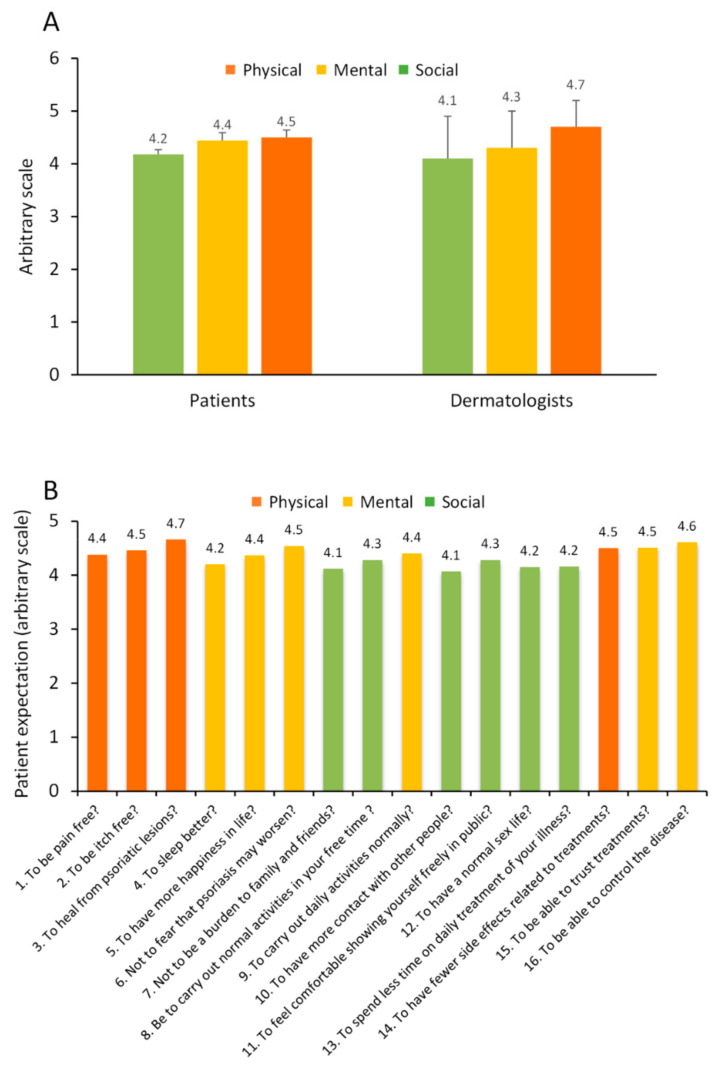
Rating of three core domains, physical, mental and social associated with achievement of patient wellbeing (“expectation”). (**A**) Perspective of patients and dermatologists on rating of three core domains associated with achievement of patient wellbeing. Data are presented as mean ± SD. (**B**) Results from questions/statements related to Question no. 23 from the online questionnaire. (**A**), Q23: With regard to Psoriasis and with specific reference to the improvement of your physical, social and emotional wellbeing, please tell us how important the following 16 statements are for you? (choose from; not at all, a little, quite a lot, very much, a lot; values from 0–5).

All 32 dermatologists completed Question 23 of the online survey in order to rate how social, physical and mental domains contribute to patient wellbeing. Using a scale from 0–5 (0 representing lowest relevance and 5 reflecting the most important), questions/issues relating to three core domains relative to patient wellbeing (i.e., physical, mental and social) were rated (Q:23 from the survey; Supplementary Materials S1). Dermatologists rated the three domains as having similar importance, although the physical component emerged as rated highest (4.7 ± 0.5), followed by mental (4.3 ± 0.7) and social (4.1 ± 0.8) with a statistically significant difference seen between physical and social domains (*p* < 0.01). These three components when rated by patients yielded similar values and followed a close pattern, highest for physical, followed by mental and social domains (Figure 4A). We next examined questions related to patient expectation (Q:23 from the online survey). Examining mean score relative to individual questions revealed similar rating when categorised for physical, mental and social domains (Figure 4B).

**Figure 5:**
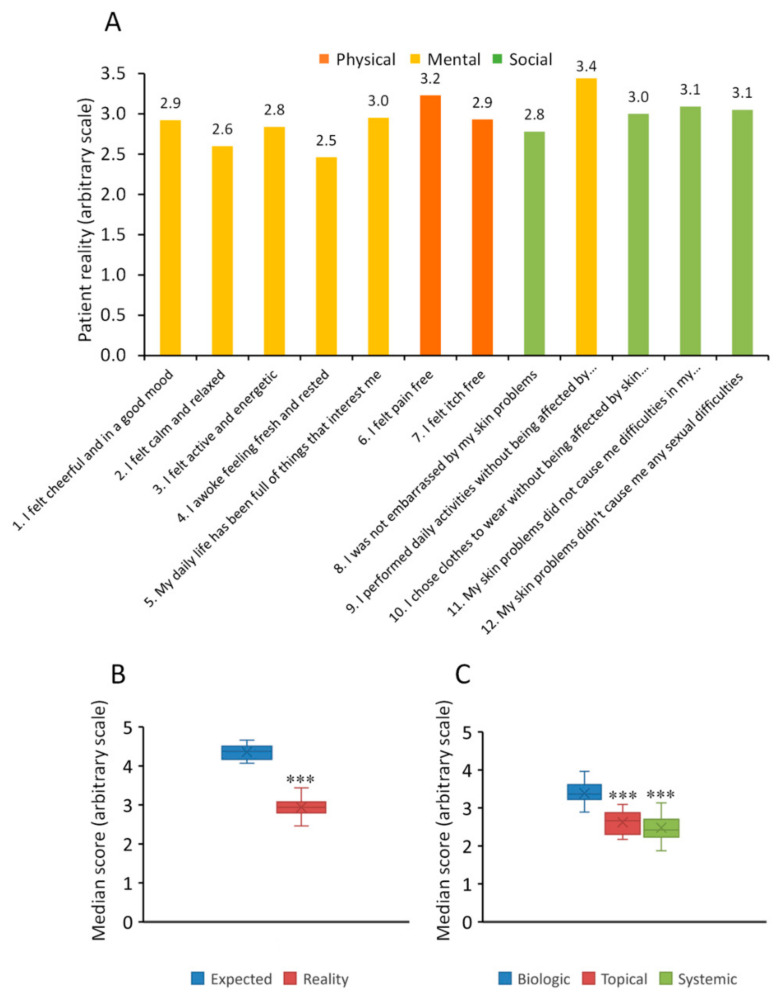
Rating of three core domains, physical, mental and social associated with achievement of patient wellbeing and the effect of different treatment. (**A**) Q24: For each of the following 12 statements, please indicate the answer that comes closest to how you have felt in the last two weeks (assign a value from 0 to 5, where 0 = never and 5 = always). Scores given to each of these questions reflect the reality that patients are experiencing. (**B**) Box-whisker plot showing the median score for their expected and reality of their psoriasis. (**C**) Box-whisker plot showing the median score stratified by treatment type. Data presented as median, 25th/75th percentiles and maximum/minimum recorded values. *** *p* < 0.0001.

We next examined questions relating to the “reality” currently experienced by the patient (Q:24 from the online survey). Individual scores ranged from 2.5–3.4 and were all substantially lower for the three areas (physical, mental and social) compared to those scores representing the reality currently experienced by the patient (Figure 5A vs. Figure 4B). Results from this analysis revealed a mean score for “expectation” of 4.4 ± 0.18 (median of 4.4; range 4.1–4.7) and that of “reality” of 2.9 ± 0.26 (median of 2.9; range 2.6–3.4), with significant difference between these scores (*p* < 0.0001) Figure 5B). Stratifying all data across the 3 domains by treatment type (biologic, topical or conventional systemic treatment) revealed that patients treated with biologics (3.4 ± 0.3) had significantly higher scores (*p* < 0.0001) compared to patients receiving topical (2.6 ± 0.29) or conventional systemic treatment (2.5 ± 0.36) (Figure 5C).

### 3.6. Output from Interactive Workshop Session

Interactive web-based workshop sessions were undertaken to explore in detail what key areas could best explain/influence the results derived from the online patient survey. Using a structured approach, with the aid an Ishikawa fishbone diagram [26], 4 specific questions (see blue boxes, Figure 2) were raised covering different areas to examine how they would address different patient types (i.e., PsO patient in general, PsO in specific locations, newly diagnosed PsO, long-standing PsO, PsO patients with comorbidities) (Figure 2). The key summary points agreed on from these workshop sessions is described below:

1. What are the most important domains to consider?

*A thorough examinatiopn* of the patient’s disease is central to understanding to what extent experience and perception affect wellbeing. The presence of comorbidities affects prognosis and may affect different life domains over time. Demographic factors can also impact upon the physical, psychological and social domains (e.g., disease duration). For the physical domain, collaboration with radiologists or rheumatologists may be necessary in patients with rheumatological involvement. For mental/social domains, collaboration with psychologists or psychiatrists may be necessary, particularly to identify depression or pathological anxiety and the use of therapies for psychological/psychiatric illness. A summary of the most important features for the three domains are summarised in Table 4.

2. What are the best scales or questionnaires to use?

*Little time* available during visits does not allow every domain to be investigated through the use of structured tools/questionnaires. Some aspects, especially those related to the psychological or working domain, can only be assessed during consultation. In clinical practice, non-quantifiable aspects are also important, such as non-verbal communication, patient clothing, feeling/empathy in the patient-physician relationship. An essential, yet often overlooked question to guide the interview during the visit is: “how are you?” or “how are you feeling today?”. It is important to trace the answer to this simple question in the medical record, even if it is generic (since it is a parameter that can also justify a possible change in therapy). To assess the physical domain, PASI [34] or body surface area (BSA) [35] can be used and the presence of joint disease can be assessed using the PEST questionnaire (psoriasis epidemiology screening tool) and the Ritchie articular index or Physical Activity Scale for the Elderly (PASE) [36]. For social and mental domains, it is mandatory to evaluate the patient’s general psychological state through simple interview questions or the Dermatology Life Quality Index (DLQI) [31], particularly to evaluate mood, depression or anxiety and current therapies for the treatment of psychological disorders. In specific cases, the complete DLQI, carried out immediately after the PASI, can be an important tool to refine the therapy or modify it. It is also important to investigate productivity and the working domain.

3. What instruments/tools do we have available in real-life practice?

*In real-life* practice in a hospital environment, there is significantly reduced time to complete questionnaires or tools to evaluate patient QoL. Simplified questionnaires (red flags) can be used to assess whether there is the presence of psoriatic arthritis or gastrointestinal problems, used in the form of an interview/questionnaire. In some cases, questionnaires can be completed prior to the visit or at home to save time. The availability of multidisciplinary teams or a collaboration with a rheumatologist is very important.

4. What therapeutic approach is needed to achieve patient wellbeing?

*Biologics* such as tumor necrosis factor (TNF), IL-12/23, IL-17 and IL-23 inhibitors have allowed dermatologists to successfully treat moderate-to-severe psoriasis.

It is necessary to consider the tailored therapy of the patient (naive, multi-failure, disease duration, major disabilities, difficult locations, comorbidities etc.). For diagnostic clarification, specialists should seek advice from other specialists, but not for the final therapeutic decision. The therapeutic approach must be considered in a view of psychological wellbeing of the patient and following careful profiling of the patient from the general medical history.

The presence of arthritis, chronic infectious disease and difficult-to-treatment locations help guide the therapeutic choice. For the young patient, effort should be made to change the course of the disease using the appropriate biological therapy; wider dosing schedules (less frequent administration) over time have a positive effect on psychological wellbeing.

In patients with comorbidities such as obesity or previous failure to different biologics, the added value of a drug is represented by the safety and the possibility to customize the dosage.

Being able to administer an effective drug, (particularly in cases of multiple comorbidities), has a positive psychological impact as the patient may avoid too many different drugs.

## 4. Discussion

This multicentre survey reflects the real-life status quo of the current routine care and management of PsO patients in Italy.

The aim of SHAPE was to obtain real-world evidence on patient perspectives on the impact of psoriasis and its treatment on patients’ daily lives and their wellbeing. We performed a two-stage structured approach; first questionnaire design and implementation and second, web-based workshop sessions to interpret and discuss results from the survey.

Results from this survey reveal that patients with moderate-to-severe psoriasis rate the three core domains associated with wellbeing (physical, mental and social) as having similar importance (also similar to rating given by dermatologists). However, based on the scores that we observe, what they desire from their treatment and their perception of the disease, strongly exceeds the reality that they are currently experiencing in terms of their treatment and perception of the disease. This disparity points towards a clear unmet need.

Patients who participated in this survey were middle-aged (52.4 years) with long-standing disease (disease duration of 22.5 years), with a high burden of comorbid diseases and widespread lesions (particularly in visible areas). Furthermore, 34 (13.5%) patients with moderate-to-severe psoriasis were not currently receiving any treatment for their psoriasis, which is unfortunately not a novel finding. The under-medication of psoriasis is well documented, often leading to worse outcome [37].

The physical burden experienced by these patients as a whole is significant and results from this survey reflect issues relating to non-physical domains such as social and mental areas are also impacted to a similar extent.

Indeed, in this respect, it is recognised that the social and psychological impact caused by psoriasis is generally underestimated by dermatologists [25,26,27] and as many as half of patients feel that their healthcare professionals do not understand the mental health impact of the disease [28]. Corroborating this finding, it also emerged from this survey, albeit to a lower extent, about 40% of patients felt that their dermatologist was not taking into consideration their wellbeing. This result may point towards a lack of communication between patient and physician. There is available evidence from other studies that the ability to communicate empathetically with patients has been shown to have a positive effect in clinical practice, in addition to establishing a trusting relationship [38]. Another study performed in Italy supports this view, showing that a dermatologist’s interpersonal skills are the most important factor likely to have a positive effect on treatment adherence and health outcomes and therefore improve patient satisfaction [39].

In the present study, the majority of patients with moderate-to-severe PsO were receiving biological treatment (42.5%), followed by topical (27.8%) and conventional systemic therapy (15.9%), a distribution that has been observed elsewhere [40,41]. We observed that patients treated with biological agents rated their treatment positively over patients treated with systemic or topical medication. The high percentage of patients with moderate-to-severe PsO receiving topical treatment reflects clinical practice: patients with severe psoriasis often arrive (even for years) without any previous treatment. Another factor that may influence treatment could be due to the fact that often these patients without previous therapies travel from rural areas where there is problem of access to treatment, Furthermore, patients with no previous therapy experience often ignore the availability of a treatment, possibly associated with communication problems in patients residing in remote areas.

Considering patients with moderate-to-severe disease, 32.6% of the patients considered the therapy they received poor/bad in relation to the improvement of signs and symptoms. This is an alarming fact. Moreover, in patients who are receiving topical treatment there is a poor acceptance of its effectiveness in improving signs and symptoms (34.3% sufficient, 37.1% poor).

The evaluation of psoriasis treatment received, in relation to the improvement of signs and symptoms, was observed to be much lower in patients on traditional systemic treatment and this could be attributed to poor tolerability rather than efficacy. Indeed, many patients may have negatively rated the therapy due to its side effects.

Patients receiving biological systemic treatment were observed to have a good if not excellent impact on their quality of life, compared to traditional systemic treatment.

The results show that both traditional systemic treatment and topical treatment have a better impact on the work domain compared with signs/symptoms and QoL. It is likely that traditional systemic treatment ensures a good impact in terms of work since it does not include tight controls. It is also possible that this specific evaluation mainly refers to the tolerability and efficacy of the therapies with regard to the critical sites that most affect the working domain (palmo-plantar areas are often the most difficult to treat).

It is necessary to make a distinction between university/hospital environments, where there are comprehensive/thorough procedures, and outpatient clinics on the territory or more peripheral, where limited time available makes it difficult to use questionnaires. Many of these patients visit centres where a DLQI or similar questionnaire is not routinely undertaken.

It should also be noted that as many as one-quarter of patients who participated in this survey had concomitant joint disease. It is recognised that psoriasis normally occurs before the development of joint symptoms [42]. However, the clinical patterns in patients with PsA can be varied and can change over time, making the recognition of the disease challenging, particularly by non-rheumatologists as well the patients themselves [43,44]. Indeed, it has been shown that PsA patients benefitting from multidisciplinary care (i.e., visited by rheumatologists and dermatologists) in a US clinic were more likely to receive systemic medication (25% vs. 15%) and be treated with a biologic agent (37% vs. 16%) than in prior care with only a dermatologist or a rheumatologist [45]. Another study recently performed in Italy also highlighted a diagnostic delay emerging from both settings with significantly different therapeutic approaches [46]. While a multidisciplinary approach may indeed be the best option for these patients [47], further surveys that address the QoL specific to patients with underlying joint disease are warranted.

Gender specific differences in patients with PsO are also recognised to exist [48]. While our preliminary analysis did not reveal any notable differences between gender, further studies with a larger sample size may yield additional information.

It is important to highlight that patients included in the present study were members of the Italian association for psoriasis (ADPISO) and this may represent potential bias as they have an extensive history and strong awareness of their disease. However, this may actually be considered to reflect a unique strength of this study since a higher proportion of patients would be expected to more accurately respond to each of the questions compared to a population where a lack of knowledge or awareness of the disease may have impacted upon their ability to provide a true and accurate estimate/reflection of the status of their disease. We believe that the homogenous nature of our population in terms of awareness and knowledge of their disease and its management should limit the degree of variability and therefore increase the precision of their response to the online questionnaire.

A study by Renzi et al. [49] found gaps in knowledge with regard to treatments in both psoriasis and psoriatic arthritis groups and this pattern has been observed elsewhere [50,51]. In an earlier study by Renzi et al. [52], it was observed that patients with good knowledge more frequently reported complete satisfaction with care compared with patients with poor knowledge. However, Lubrano et al. [50], observed a significant association between educational level and knowledge. It is plausible that individuals with a higher educational level are more interested in acquiring knowledge. Furthermore, people with more severe disease may experience the importance of medical knowledge in order to keep their symptoms under control.

Given the fact that all patients had received at least primary and middle school education (up to 14 years in Italy) and 86.2% of patients attended at least secondary school or university (slightly higher in moderate-to severe patients), their educational level coupled with their knowledge of psoriasis through the ADIPSO association should have helped to “standardise” the quality of their response.

## 5. Strengths and Limitations

The strengths of this survey lie in the real-life, multiregional, cross-sectional design where patients could voluntarily provide important insight on their wellbeing status as well as their levels of satisfaction and their perceptions of how they are being currently treated.

The proportion of patients who completed the survey was not equally distributed across macroareas. However, a total of 392 patients across different geographic areas of the country can be considered representative of the Italian territory. The online survey was based on the PBI, DLQI and WHO-5 wellbeing index comprising 26 questions to cover issues and areas that could impact on patients QoL and wellbeing. The questionnaire was not validated but it was based on 3 validated questionnaires. It was also designed by 4 experts who have extensive experience in the management of patients with psoriasis and a particular interest in the wellbeing and QoL of patients with this disease. For some of the questions in the survey, a small number of patients (*n* = 10) did not respond to questions or may not have known the answer. These patients were elderly (all were >75 years) and did not suffer physical disability. These patients may have also lacked the basic computer skills to be able to complete the questionnaire online and in these cases sought help from ADIPSO. We agree this may represent potential bias. Another limitation was that patients evaluated the severity of their disease themselves and this may not reflect the reality as judged by a dermatologist. Last, since this was an epidemiological study, causality was not assessed.

## 6. Conclusions

In this survey, we identified key components contributing to barriers impacting on the wellbeing in patients with moderate-to-severe PsO. An important disparity with regard to “expected” and “reality” for aspects/items relating to wellbeing was highlighted. Approximately 40% of patients felt that their dermatologist was not considering their wellbeing and a similar proportion felt that their current treatment was inadequate for improving signs and symptoms. Prioritising patient’s QoL can lead to a more targeted and tailored treatment. When a lack of time can impact on the doctor-patient relationship, having a simple, but comprehensive questionnaire can facilitate treatment choice and reduce patient delays. In routine clinical practice, dermatologists can never underestimate the importance of the simple question: “how are you feeling today?”.

## Figures and Tables

**Table 1:** Clinical characteristics of psoriasis patients.

Clinical Characteristic	All PsO Patients * (*n* = 392)	Moderate-to-Severe PsO (*n* = 252; 64.3%)	Mild PsO (*n* = 73; 18.6%)	*p*-Value
*General*				
Male gender, *n* (%)	210 (53.6)	143 (56.8)	36 (49.3)	0.32
Age (years)	52.4 ± 14.8	53.7 ± 13.5	49.01 ± 17.5	**0.017**
BMI (kg/m^2^)	26.1 ± 4.3	26.5 ± 4.4	25.1 ± 4.3	**0.015**
Disease duration	22.5 ± 14	23.6 ± 14	21.2 ± 14.2	0.21
*Education*				
Primary	54 (13.8)	31 (12.3)	8 (10.9)	0.92
Secondary (high school)	218 (55.6)	143 (56.8)	42 (57.6)	0.91
University degree	120 (30.6)	78 (30.9)	23 (31.5)	0.93
*Civil status*				
Student	20 (5.1)	7 (2.8)	8 (10.9)	**0.009**
Employed	220 (56.1)	150 (59.5)	40 (54.8)	0.56
Pension/retired	96 (24.5)	65 (25.8)	13 (17.8)	0.21
Unemployed	56 (14.3)	30 (11.9)	12 (15.1)	0.41
*Lesion localization*				
Elbow/knee	251 (70.1)	182 (72.2)	45 (61.6)	0.11
Scalp	228 (63.7)	167 (66.3)	39 (53.4)	0.062
Chest	179 (50)	147 (58.3)	17 (23.3)	<**0.0001**
Hands/feet	138 (38.6)	119 (47.2)	12 (16.4)	<**0.0001**
Nails	126 (35.2)	106 (42.1)	11 (15.1)	<**0.0001**
Genitals	85 (23.7)	66 (26.2)	11 (15.1)	0.07
Face	77 (21.5)	61 (24.2)	10 (13.7)	0.08
*Comorbidities*, *n (%)*				
Hypertension	99 (27.7)	75 (29.8)	14 (19.2)	0.1
Rheumatological disease	92 (25.7)	69 (27.4)	11 (15.1)	**0.046**
Gastrointestinal disorder	46 (12.9)	32 (12.7)	11 (15.1)	0.74
Obesity	38 (10.6)	32 (12.7)	5 (6.9)	0.24
Depression	38 (10.6)	31 (12.3)	5 (6.9)	0.27
Cardiovascular disease	29 (8.1)	21 (8.3)	7 (9.6)	0.92
No other pathologies	106 (29.6)	67 (26.6)	29 (39.7)	**0.043**

BMI = body mass index, Data presented as mean ± standard deviation or number and %. * A total of 67 patients did not know or did not respond to the question regarding “which type of psoriasis were you diagnosed with?”, i.e., missing values. Statistically significant *p*-values are shown in bold text.

**Table 2:** Previous and current treatment.

Clinical Characteristic	All PsO Patients *n* = 392 *	Moderate-Severe PsO *n* = 252	Mild PsO (*n* = 73)	*p*-Value
*Previous treatment n*, (%)				
Topical	272 (75.9)	192 (76.2)	56 (76.7)	0.93
Systemic therapy	69 (19.3)	53 (21)	9 (12.3)	0.13
≥1 systemic therapy	115 (32.1)	103 (40.9)	7 (9.6)	<**0.0001**
Biological treatment	32(8.9)	26 (10.3)	5 (6.9)	0.51
≥1 biological treatment	31 (8.7)	27 (10.7)	2 (2.7)	0.06
No previous therapy	23 (6.4)	12 (4.8)	8 (10.9)	0.09
*Current treatment n*, (%)				
Topical	124 (34.6)	70 (27.8)	47 (64.4)	<**0.0001**
Systemic therapy	49 (13.7)	40 (15.9)	6 (8.2)	0.14
Biological treatment	118 (32.9)	107 (42.5)	3 (4.1)	<**0.0001**
No treatment	61 (17)	34 (13.5)	16 (21.9)	0.12

Data presented as number and %. * A total of 67 patients did not know or did not respond to the question regarding “which type of psoriasis were you diagnosed with?”, i.e., missing values. Statistically significant *p*-values are shown in bold text.

**Table 3:** Summary of results derived from survey questions related to the impact of psoriasis treatment on signs and symptoms, quality of life and work.

	All PsO Patients	Moderate-to-Severe PsO		
Question and Rating	All Therapy (*n* = 252) *	Biologicals (*n* = 107)	Systemic (*n* = 40)	Topical (*n* = 70)
**Question 18.** How do you rate the psoriasis treatment you have received in recent years in relation to the improvement in signs and symptoms?				
Great	52 (20.6)	45 (42.1)	1 (2.5)	2 (2.9)
Good	58 (23.0)	40 (37.4)	4 (10)	11 (15.7)
Enough/sufficient	56 (22.2)	18 (18.8)	9 (22.5)	24 (34.3)
Poor	66 (26.2)	4 (3.7)	25 (62.5)	26 (37.1)
Bad	16 (6.4)	0 (0)	1 (2.5)	7 (10)
Doesn’t know/no answer	4 (1.6)	0 (0)	0 (0)	0 (0)
Total	252 (100)	107 (100)	40 (100)	70 (100)
**Question 19.** How do you rate the psoriasis treatment you have received in recent years, in relation to the improvement of your quality of life (social relationships, psychological state)?				
Great	43 (17.1)	37 (34.6)	1 (2.5)	3 (4.3)
Good	69 (27.4)	47 (43.9)	5 (12.5)	9 (12.9)
Enough/sufficient	63 (25)	19 (17.8)	13 (32.5)	26 (37.1)
Poor	56 (22.2)	4 (3.7)	19 (47.5)	21 (30.0)
Bad	16 (6.4)	0 (0)	2 (5.0)	10 (14.3)
Doesn’t know/no answer	5 (1.9)	0 (0)	0 (0)	1 (1.4)
Total	252 (100)	107 (100)	40 (100)	70 (100)
**Question 20.** How do you rate the psoriasis treatment you have received in recent years, in relation to its impact on the workplace?				
Great	40 (15.9)	35 (32.7)	1 (2.5)	1 (1.4)
Good	55 (21.8)	38 (35.5)	4 (10)	8 (11.4)
Enough/sufficient	54 (21.4)	20 (18.7)	9 (22.5)	21 (30.0)
Poor	58 (23.0)	5 (4.7)	17 (42.5)	25 (35.7)
Bad	8 (3.2)	0 (0)	2 (5.0)	3 (4.3)
Doesn’t know	2 (0.8)	1 (0.9)	0 (0)	0 (0)
Not relevant in my case	35 (13.9)	8 (7.5)	7 (17.5)	12 (17.1)
Total	252 (100)	107 (100)	40 (100)	70 (100)

Data are presented as number of patients who responded to these specific questions expressed as a percentage. * 34 patients were currently not receiving any treatment and for patients with moderate-to-severe psoriasis and 1 patient did not know or did not respond.

**Table 4:** The most important domains (physical, social and psychological) to consider—output from interactive workshop session.

Physical Domain	Mental and Social Domains
Disease duration disease severity (*red flags that may signify a level of systemic inflammation that could increase the risk of cardiovascular disease*)	Comorbidities (*be careful to differentiate depression as a psychiatric comorbidity and depression understood as “complaining”*).
2. Type of psoriasis (*chronic, stable, plaque, pustular form etc.*)	2. Possible concomitant therapies for psychological/psychiatric illness (*e.g., antidepressants*)
3. Number of relapses	3. Attention given to anger and related behavioural signs (*anger and rage are often associated with therapies, e.g., the patient treated with topical drugs rather than systemic or biological therapies*)
4. Lesions in visible and sensitive areas (*scalp, face, hands, genitals*) *and specific sites of involvement* (*genital areas, intergluteal areas, hands*)	4. Productivity and work
5. Itching symptoms	5. Social domain and psychosocial discomfort associated with age (*young patients have more marked psychosocial discomfort*)
6. Pain (*skin and joint*)	6. Physical symptoms that affect the psychological and social domains
7. Presence of psoriatic arthritis	7. Inability to lead a normal life (*sleep, leisure time, sports activity, patient’s sex life*)
8. Comorbidities (*for newly diagnosed psoriasis, is it is important to be aware of other therapies (e.g., cancer patients or patients with inflammatory bowel disease*)	8. Negative side effects caused by therapies
	9. Trust in therapy (*important to discuss duration of therapies with the patient*)

## Data Availability

Data can be made available from the corresponding author upon request.

## Supplementary Materials

The following supporting information can be downloaded at: https://www.mdpi.com/article/10.3390/jcm11102801/s1, Supplementary Materials S1: Online survey available from ADIPSO website (in Italian): https://it.research.net/r/benesserepaziente (accessed on 12 November 2021); Supplementary Materials S2: Development of an online questionnaire based on the WHO-5, DLQI and PBI questionnaires.
