# Supplementary material for: Sharing Patient and Clinician Experiences of Moderate-to-Severe Psoriasis: A Nationwide Italian Survey and Expert Opinion to Explore Barriers Impacting upon Patient Wellbeing
Source: J Clin Med. 2022 May 16;11(10):2801. doi: 10.3390/jcm11102801 (PMC9146704; doi:10.3390/jcm11102801)
Supplement: Supplementary file 1 [file jcm-11-02801-s001.zip › Supplementary Material S1.pdf]

Online survey available from ADIPSO website (in Italian):
https://it.research.net/r/benesserepaziente

SOCIO-DEMOGRAPHIC

1. **Age** (specify year)
2. **Sex** (male or female)
3. **If female**; number of pregnancies, number of stillborn births, age of first menstrual cycle
4. **Anthropometric measures**; (specify weight and height)
5. **Region where resident**; (choose from one of 20 regions in Italy)
6. **Education**; obligatory School diploma (elementary school and lower middle school), high school graduation or degree
7. **Employment status**; (specify whether student, employed, retired or unemployed)

INFORMATION ON PSORIASIS DISEASE

8. **What type of psoriasis have you been diagnosed with?** (mild, moderate, severe or doesn't know/doesn't answer)
9. **What are the affected areas?** (trunk, face, scalp, hands/feet, genitals, elbows/knees or nails)
10. **In what year were you diagnosed with psoriasis?** (specify the year)
11. **If you remember, please also enter the month** (specify the month)
12. **How long has it been since you were diagnosed with psoriasis before starting psoriasis specific therapy?** (indicate the number of months)
13. **Are you currently taking specific treatment for psoriasis?** (choose from; yes, biological systemic treatment, yes, traditional systemic treatment, yes, topical treatment, no, no treatment, doesn’t know/doesn't answer)
14. **What kind of treatments, other than the current one, have you previously taken for Psoriasis?** (choose from; a systemic biological treatment, more than one systemic biological treatment, a traditional systemic treatment, more than one traditional systemic treatment, topical treatments, no previous treatment)
15. **Do you have any other pathologies besides Psoriasis?** (choose whatever disease is appropriate from; pneumopathies: asthma, chronic obstructive diseases, interstitial diseases; cardiovascular diseases: angina/arrhythmias, heart attack; hypertension, stroke, diabetes mellitus, rheumatological diseases: arthritis, osteoarthritis, osteoporosis; gastrointestinal disorders: gastropathies, liver and intestinal diseases; neoplasms, obesity, kidney failure, depression, fibromyalgia, transplant, no other pathology besides psoriasis, other)
16. **Do you have other family members diagnosed with Psoriasis?** (choose no or yes, if yes, please specify who)
17. **Have you always been treated at the same center/with your current dermatologist in the last three years?** (choose from; yes, no, doesn't know / doesn't answer)
18. **How do you rate the psoriasis treatment you have received in recent years in relation to the improvement in signs and symptoms?** (choose from; great, good, enough, poor, bad, doesn't know / doesn't answer)
19. **How do you rate the psoriasis treatment you have received in recent years, in relation to the improvement of your quality of life (social relationships,**

psychological state)? (choose from; great, good, enough, poor, bad, doesn't know/doesn't answer)

20. **How do you rate the psoriasis treatment you have received in recent years, in relation to its impact on the workplace?** (choose from; great, good, enough, poor, bad, doesn't know, not applicable in my case)
21. **How much do your family members help and support you in dealing with your life with psoriasis?** (choose from; very much, enough, a little, not at all, doesn't know/doesn't answer)
22. **Have you ever filled out a questionnaire or an assessment scale to measure how your pathology impacts on your quality of life (e.g. daily activities that you are more or less able to carry out, impact of the disease on social relationships, psychological distress, ...)?** (choose from yes or no, doesn't know/doesn't answer)
23. **With regard to Psoriasis and with specific reference to the improvement of your physical, social and emotional well-being, please tell us how important the following 16 statements are for you?** (choose from; not at all, a little, quite a lot, very much, a lot)

    1. How important is it to you to be pain free?
    2. How important is it to you to be itch free?
    3. How important is it for you to heal from psoriatic lesions?
    4. How important is it for you to sleep better?
    5. How important is it for you to have more joy to live?
    6. How important is it for you to be able to feel free from the fear that psoriasis may get worse?
    7. How important is it for you not to be a burden to family and friends?
    8. How important is it for you to be able to carry out normal activities in your free time (activities with friends, family, or sports)?
    9. How important is it for you to be able to carry out daily activities normally (shopping / looking after the house or garden or working or studying)?
    10. How important is it for you to be able to have more contact with other people?
    11. How important is it for you to feel comfortable showing yourself freely in public?
    12. How important is it for you to be able to have a normal sex life?
    13. How important is it for you to spend less time on daily treatment of your illness?
    14. How important is it for you to have fewer side effects related to treatments?
    15. How important is it for you to be able to trust treatments?
    16. How important is it for you to be able to control the disease?

24. **For each of the following 12 statements, please indicate the answer that comes closest to how you have felt in the last two weeks** (assign a value from 0 to 5, where 0 = never and 5 = always)

    1. I felt cheerful and in a good mood
    2. I felt calm and relaxed
    3. I felt active and energetic
    4. I woke up feeling fresh and rested
    5. My everyday life has been full of things that interest me
    6. I felt pain free
    7. I felt itch free
    8. I felt free from the embarrassment or discomfort caused by my skin problems

9. I carried out my daily activities (shopping / caring for the house or garden or working or studying) without being affected by my skin problems
10. I chose the clothes to wear without being affected by my skin problems
11. My skin problems did not cause me difficulties in my free time (activities with friends, family, or sports)
12. My skin problems didn't cause me any sexual difficulties

25. **How do you rate the psoriasis treatment you have received in the past year in relation to maintaining the benefit over time and your satisfaction?** (choose from; great, good, enough, poor, bad, doesn't know/doesn't answer)

26. **How much do you think the aspects of your pathology relating to the Quality of Life (work environment, social relationships, psychological state, ...) are taken into consideration by the dermatologist?** (choose from; not at all, a little, quite a lot, very much, a lot, doesn’t know how to respond or doesn’t respond)
