# Supplementary material for: Sharing Patient and Clinician Experiences of Moderate-to-Severe Psoriasis: A Nationwide Italian Survey and Expert Opinion to Explore Barriers Impacting upon Patient Wellbeing
Source: J Clin Med. 2022 May 16;11(10):2801. doi: 10.3390/jcm11102801 (PMC9146704; doi:10.3390/jcm11102801)
Supplement: Supplementary file 1 [file jcm-11-02801-s001.zip › Supplementary Material S2.pdf]

Supplementary Material S2. Development of an online questionnaire based on the WHO-5, DLQI and PBI questionnaires.

The main differences in our questionnaire compared to DLQI, WHO-5 and PBI was that questions were wide ranging in terms of the questions asked with particular emphasis placed on awareness of the disease linked to the experience of dermatologists in a real-life setting treating patients with this chronic disease.
Our aim was to develop an online questionnaire that encompasses current perception (their reality) in a single questionnaire that could be performed quickly and easily.
The survey "photographs" the patient's perception of specific aspects related to well-being in daily real-life while also highlighting "expectation vs. reality".

Our survey questionnaire was developed in order to collect information on the following areas:

1. Socio-demographic data (age, sex, anthropometric measures, educational qualifications, employment status etc.; Questions 1-7)
2. Disease characteristics (type of psoriasis diagnosed, current and previous therapy, patient history etc.; (Questions 8-22)
3. Data on aspects/items related to well-being from the patient's point of view (Questions 23-26)

Points 1 and 2 were not linked or based on a questionnaire and were developed by the Authors, whereas Point 3 was based on the DLQI, WHO-5 and PBI.

The importance that the patient attributes to the individual aspects/items that make up the 3 core domains of well-being (physical, social, mental/emotional), form an important component of the questionnaire. Q23 of the questionnaire contains 16 statements to evaluate the expectation of the patient with regard to physical, social and mental/emotional domains based on 25 questions from the first part of the Patient Benefit Index (PBI) questionnaire ("As a result of therapy, how important is it for you to.."). The Patient Benefit Index, which is a validated instrument for assessing patient-relevant benefit in skin diseases and best suited for the purpose that we wanted to achieve with the questions evaluating patient's "expectations".
Q24 from the survey contain 12 statements evaluating patient "reality" in the past 2 weeks. The first 5 statements were based on the WHO-5 questionnaire and statements 6-12 were based on the DLQI as well as the second part of the PBI ("The current treatment has helped me to.."). These three validated questionnaires were considered the most effective instruments to capture the information from patients regarding patient wellbeing in the psoriasis setting.
Questions 25 and Q26 were developed by the Authors.

Augustin, M.; Radtke, M.A.; Zschocke, I.; Blome, C.; Behechtnejad, J.; Schäfer, I.; Reusch, M.; Mielke, V.; Rustenbach, S.J. The Patient Benefit Index: A Novel Approach in Patient-Defined Outcomes Measurement for Skin Diseases. *Arch Dermatol Res* **2009**, *301*, 561–571, doi:10.1007/s00403-009-0928-8.

Finlay, A.Y.; Khan, G.K. Dermatology Life Quality Index (DLQI)--a Simple Practical Measure for Routine Clinical Use. *Clin. Exp. Dermatol.* **1994**, *19*, 210–216.

Bech, P. Clinical Psychometrics | Wiley Available online: https://www.wiley.com/en-us/Clinical+Psychometrics-p-9781118329788 (accessed on 2 December 2021).

Topp, C.W.; Østergaard, S.D.; Søndergaard, S.; Bech, P. The WHO-5 Well-Being Index: A Systematic Review of the Literature. *Psychother Psychosom* **2015**, *84*, 167–176, doi:10.1159/000376585.
